# Increasing health worker capacity through distance learning: a comprehensive review of programmes in Tanzania

**DOI:** 10.1186/1478-4491-8-30

**Published:** 2010-12-31

**Authors:** Anya J Nartker, Liz Stevens, Alyson Shumays, Martin Kalowela, Daniel Kisimbo, Katy Potter

**Affiliations:** 1 International Training and Education Center for Health, Department of Global Health, University of Washington, Seattle, USA; 2 International Training and Education Center for Health, Dar es Salaam, Tanzania; 3 Ministry of Health and Social Welfare, Centre for Distance Education, Morogoro, Tanzania

## Abstract

**Background:**

Tanzania, like many developing countries, faces a crisis in human resources for health. The government has looked for ways to increase the number and skills of health workers, including using distance learning in their training. In 2008, the authors reviewed and assessed the country's current distance learning programmes for health care workers, as well as those in countries with similar human resource challenges, to determine the feasibility of distance learning to meet the need of an increased and more skilled health workforce.

**Methods:**

Data were collected from 25 distance learning programmes at health training institutions, universities, and non-governmental organizations throughout the country from May to August 2008. Methods included internet research; desk review; telephone, email and mail-in surveys; on-site observations; interviews with programme managers, instructors, students, information technology specialists, preceptors, health care workers and Ministry of Health and Social Welfare representatives; and a focus group with national HIV/AIDS care and treatment organizations.

**Results:**

Challenges include lack of guidelines for administrators, instructors and preceptors of distance learning programmes regarding roles and responsibilities; absence of competencies for clinical components of curricula; and technological constraints such as lack of access to computers and to the internet. Insufficient funding resulted in personnel shortages, lack of appropriate training for personnel, and lack of materials for students.

Nonetheless, current and prospective students expressed overwhelming enthusiasm for scale-up of distance learning because of the unique financial and social benefits offered by these programs. Participants were retained as employees in their health care facilities, and remained in their communities and supported their families while advancing their careers. Space in health training institutions was freed up for new students entering in-residence pre-service training.

**Conclusions:**

A blended print-based distance learning model is most feasible at the national level due to current resource and infrastructure constraints. With an increase in staffing; improvement of infrastructure, coordination and curricula; and decentralization to the zonal or district level, distance learning can be an effective method to increase both the skills and the numbers of qualified health care workers capable of meeting the health care needs of the Tanzanian population.

## Background

Tanzania, like many other developing countries, faces a crisis in human resources for health. It has a population of 40 million, 75% of which lives in rural areas in the 21 regions on the mainland; in Zanzibar, this figure is 60%. Tanzania is one of the poorest countries in the world, with a per capita income of US$ 400. HIV prevalence is at 6%, and the average life expectancy is 51 years [1]. These population characteristics impact the health care system in several ways, including an ever-increasing need for skilled health care workers willing to work in remote rural areas.

The Tanzania Ministry of Health and Social Welfare (MoHSW) estimated that as of 2006, the health care system was operating with a 65% shortage of the required skilled workforce [1]. In addition, the MoHSW has launched a ten-year programme to ensure that all Tanzanians have access to health care services. This programme--the *Mpango wa Maendeleo wa Afya ya Msingi *(MMAM), or Primary Health Services Development Programme (PHSDP)--is intended to expand and improve the provision of health services to the level of every village and every ward. Meeting this mandate will create the need for even more qualified health care workers, with a goal of training 460 000 new health care workers by 2017 [2]. This goal assumes a stable workforce, but retention of health care workers, particularly in remote settings, is difficult. In addition, upgrading the qualifications and skills of the current health workforce is challenging, in terms of cost and accessibility of training, and in absenteeism from work as a result of attending training programmes in other locations.

The Tanzania MoHSW has tried to address these challenges by providing upgrading programs for health care workers utilising distance learning. In 1998, the MoHSW created the Centre for Distance Education (CDE) to serve as the national coordinating centre for distance learning programmes for health care workers in Tanzania. The CDE offers three in-service upgrading programmes for health care workers: Clinical Assistant to Clinical Officer, Maternal Child Health Aide to Enrolled Nurse, and Enrolled Nurse to Registered Nurse. Total enrolment is over 1500 students, with 160 graduates to date.

In addition to the CDE, several other distance learning programmes operate in Tanzania. They include other health care worker upgrading programmes that utilise print and computer technology, continuing education programmes that broadcast nationally and internationally using videoconferencing technology, e-learning courses on HIV/AIDS and other healthcare topics, and telemedicine projects, to name a few.

In 2008, the MoHSW and its partners undertook an assessment of the CDE, as well as several other distance learning programmes in Tanzania [3]. The object of the study was to determine the feasibility and success of distance learning programmes in Tanzania and their ability to help Tanzania meet its human resources for health (HRH) needs. The findings can be applicable to other countries and resource-limited settings considering implementing distance learning programs as a national strategy to address gaps in HRH.

### Health care worker training, retention, and distance learning

Several studies support the use of health care worker in-service training, qualifications upgrading, and post graduate training as a way to motivate and retain health care workers. A review of 16 countries in east and southern Africa found that most offer training and career path development as one of several non-financial incentives for the retention of health care workers [4]. Willis-Shattuck, et al., in a systematic review of 20 articles focused on Africa and Asia, found that career development and continuing education were motivational factors for health care workers [5]. Matheur and Imhoff interviewed health care workers in Benin and Kenya and found that training was an important motivator [6]. Respondents mentioned that following training, they often felt more confident and felt increased commitment and interest in their work. However, many noted that training must be relevant to the local context and reflect actual working conditions. Awases, et al, surveyed 2383 health professionals in six countries regarding health care worker migration [7]. They analyzed several factors that would encourage health care workers to stay in their home countries. Having opportunities for accessible continuing education and training was cited by a majority of respondents in each country, and in South Africa, many respondents mentioned "innovative training opportunities such as distance education" as a motivator. Although all the study countries reported some available training opportunities, health professionals working in rural areas were often left out.

White, et al., found that continuing medical education opportunities are perceived by doctors practicing in remote settings to increase confidence, alleviate professional isolation, and that access to these opportunities is a factor in health care workers remaining in rural practice in remote locations [8]. Kotzee and Couper surveyed South African doctors in rural settings, and they reported that access to continuing medical education, post-graduate upgrading, short courses, and internet access for distance education were important factors in retention [9]. In another study in Tanzania, Manongi found that in remote and rural areas with limited staff, many health care workers are being called to handle cases for which they are not trained [10]. Health care workers in these settings saw the solution as not hiring more qualified staff: they wanted more training for themselves in order to perform their expanded jobs competently.

However, these studies also noted challenges involved in the training of health care workers. As more initiatives are developed to train health care workers to respond to complex diseases like HIV/AIDS and malaria, it means that health care workers are removed from their posts for a long time to attend multiple trainings, increasing the burden on an already overwhelmed system [10].

Several articles offer distance learning as a solution to health care worker training challenges. Distance learning is learning that takes place with the teacher and learner in physically separate locations. Interaction occurs through one or more types of media that can be as basic as print-based distance learning, or as advanced as computer-based (e-learning) or internet-based (on-line) distance learning. Médecins Sans Frontières cited the creation of post-graduate distance learning courses as a way to alleviate absenteeism from clinical work but still allow health care workers to receive needed training [11]. Kinfu, et al., in an article about health care worker shortages and migration in Africa, suggested telemedicine as one way to reduce the health care worker outflow [12]. Knebel, in a review of over 100 articles about distance learning in health care, found that the major benefit of distance learning was the convenience and accessibility of training for those who do not live near traditional training centres and universities [13]. This is particularly true of health care workers in rural settings, who, through distance learning, can still receive training without interruption of health care delivery and without loss of salary or negative impact on family life.

In addition, there are limited resources in developing countries to expand traditional education: lack of funds, lack of teachers, and poor infrastructure. Governments see distance learning as a cheaper way to educate more people. Knebel also cited distance learning as a way to stem the brain drain to foreign educational institutions. Other authors also point to the advantages of distance learning to broaden access to training and to decrease costs. An MPH programme at the University of Western Cape in South Africa provides degrees to health professionals in 20 other African countries while they continue to remain at their posts. The completion rate prior to the start of the distance learning program in 2000 was 33%; completion rates for the three public health qualifications offered via distance learning from 2000-2007 ranged from 57% to 72%. In addition, the completion rates over that seven-year period showed a dramatic improvement as distance learning experience grew and challenges were addressed [14].

While several studies have found distance learning to be an effective way to train health care workers [15,16], there is very little rigorous research comparing distance learning to more traditional classroom teaching modalities [13,17,18] and a lack of studies on cost effectiveness. Most studies compared test results, evaluated student satisfaction, or used student self assessment to measure their change in skills and knowledge. Others were case reports and programme descriptions. There is a dearth of published studies that followed-up students in a clinical setting after graduation to see how they performed compared to their residential programme counterparts. In addition, most research in this area did not use random selection, nor were measures tested for reliability and validity, and often, confounding variables were not taken into account.

## Methods

### Study design

This exploratory assessment was carried out during May-August 2008 and involved the use of various methods, including: internet research, a desk review, written questionnaires, telephone and e-mail surveys, a focus group, structured interviews and on-site observations. In addition, members of the assessment team attended two conferences in Tanzania related to distance learning.

### Preliminary research

Assessment staff conducted preliminary research to identify current distance learning programmes in Tanzania through internet research and networking with training partners and with the MoHSW. Initial contact was made with these programmes via e-mail and telephone. When feasible, pre-assessment visits were carried out to acquire background information on the programmes, to establish relationships with respondents prior to the actual assessment, and to schedule the assessment team visits. A total of 25 programmes in Tanzania were identified for the assessment team to visit.

In addition to collecting information on distance learning programmes in Tanzania, a thorough desk review was conducted to gather information on distance learning activities from organisations working in countries with contexts similar to that of Tanzania. Websites, programme reports, and published articles were reviewed for nine organisations conducting distance learning programmes. A programme-level survey was also used to collect information via e-mail and telephone from 13 organisations. The survey focused on each organisation's background, programmatic challenges and strengths, technological constraints, and future vision. Data from the desk review, surveys, and site visits were compiled to create a distance learning inventory, which includes a total of 49 programmes.

### Data collection

Site visits were conducted with twenty-five distance learning programmes across eight locations: Arusha, Dar es Salaam, Kigoma, Kilosa, Maswa, Morogoro, Mwanza and Zanzibar. To obtain a diverse view of the programmes' challenges and successes, on-site data were collected from programme managers, tutors, students, and information technology (IT) specialists through both structured interviews and questionnaires (see Table 1). Structured interviews were conducted with distance learning programme managers to capture information on the achievements and challenges of the programme, future plans, and how the programme addresses health care worker shortages in Tanzania.

**Table 1:** Data collection methods

Method	Target group	Number of informants
Structured interviews	Programme managers	22

Structured interviews (6) Questionnaires (2)	Distance learning tutors	8

Structured interviews (20) Questionnaires (14)	Distance learning students	34

Structured interviews	IT specialists	9*^a^*

Structured interviews	Distance learning preceptors	4

Structured interviews	Health decision makers (MoHSW)	2

Mail-in surveys	Health care workers	46

Focus group discussion (1)	HIV & AIDS care and treatment partners (training organisations)	7 (1 from each organisation)

*^a^*This number includes both IT support staff at distance learning programmes visited and IT specialists at internet service provider firms visited.

Tutors and students were interviewed to obtain their insights on student/tutor interactions, practicum components, curriculum and course materials, and how students planned to apply their new knowledge and skills after completing the programme. While structured interviews were the primary means used to collect information from students and tutors during the site visits, in some cases questionnaires were distributed because of time and/or resource constraints. The questionnaires posed identical questions in the same order as the interview guides. Although it would have been preferable to conduct interviews with every respondent, the questionnaires allowed the assessment teams to gather the same information from a greater number of individuals.

In addition, interviews with information technology (IT) specialists were conducted to gain an understanding of the technological context and to assess the feasibility of implementing various distance learning technologies and digital videoconferencing in Tanzania. Specialists were interviewed about internet and telecommunications connectivity, electrical power, and technology access issues.

Data were also gathered through on-site observations of distance learning activities in order to understand the constraints and opportunities of programmes as well as the technologies utilised in the distance learning programmes.

To triangulate the information collected during observations and site visits, data were collected from additional sources in Tanzania (see Table 1). Target groups included distance learning preceptors, health care decision makers, health care workers, and HIV and AIDS care and treatment partners. Distance learning preceptors were targeted to capture their perspectives on challenges and achievements of the practical or clinical components of distance learning programmes. To gain insight into the training needs of health care workers in Tanzania and to determine whether distance learning could meet those needs, health decision makers in different branches of the MoHSW were interviewed using a structured interview guide. Health care workers were also targeted to learn about their training needs, as well as their interest in and level of experience with distance learning programmes. Surveys used for this purpose included both open-ended and closed questions, which were distributed to respondents and returned to I-TECH by post.

Finally, a focus group discussion was held with seven participants from key HIV and AIDS care and treatment training organisations in Tanzania. The discussion was conducted to obtain additional viewpoints on the training needs of health care workers in Tanzania and to determine whether partners believed distance learning could help meet those needs. Information was also solicited on the participants' experiences with distance learning (See Table 2).

**Table 2:** Key questions addressed by methods

Method	Feasibility of distance learning	Success of distance learning programs	Ability to address HRH issues
Desk review/Internet search	X		

Structured interviews	X	X	X

Focus group	X	X	X

Mail-in surveys		X	X

Observations	X	X	

Seven assessment team members were trained in data collection and use of the assessment tools for one day. They were grouped into two teams of 3-4 members each, and each team visited 12-13 sites.

All respondents were informed as to the purpose of the assessment, the ways in which the data would be used, and that their responses would remain anonymous. All interview and focus group participants provided verbal informed consent. This assessment was approved by the U. S. Centers for Disease Control and Prevention Global AIDS Program office in Tanzania and by the MoHSW.

### Description of sampling

This exploratory assessment aimed to capture data across a broad spectrum of distance learning programmes for health care workers. 'Distance learning programme' was defined as any programme where learning takes place with the teacher and learner in physically separate locations, regardless of media type. All distance learning programmes that met this definition and that served health care workers in Tanzania and comparable resource-limited settings were considered in the assessment. Additional programmes not specific to health care workers were also included if it was believed they could provide useful data to improve distance learning programmes for health care workers. A total of 25 distance learning programmes in Tanzania were included in the assessment.

Respondents were sampled using both purposive and convenience sampling for this assessment. Health decision makers and HIV and AIDS care and treatment partners were purposively selected to gain insight into the training needs of health care workers and to determine whether distance learning could meet those needs. Programme managers, tutors, students, health care workers, preceptors, and IT specialists were convenience sampled in order to maximize the number of possible respondents.

### Data analysis

Qualitative data from interviews with preceptors and health decision makers and qualitative data from the focus group were typed up in Microsoft Word, checked for accuracy and coded by hand. Qualitative and quantitative data from the health care worker surveys, and interviews and questionnaires with programme managers, tutors, students, and IT specialists were entered in Microsoft Excel. A data quality assurance check was conducted by two staff members to ensure completeness and accuracy. General themes and their associated codes were agreed upon by the data analysis team and thematic coding was used to analyze all qualitative data, according to assessment objectives. Simple tabulation was used to analyze quantitative data in Microsoft Excel.

## Results

The assessment team found that a good foundation for distance learning exists, with a surprising number of distance learning programmes operating in Tanzania and in the region. These varied from low-tech print-based programmes such as the upgrading programmes operated by the CDE to high-end international video-conferencing operated by the Tanzania Global Development Learning Centre. A variety of other programmes exist, including HIV/AIDS-related e-learning modules such as International Weiteribidiung unde Entwicklung gGmb's (InWEnt) Global Campus 21 and WHO's IMAI Computerised Adaptation and Training Tool, as well as low-end internet-based videoconferencing and web casting operated by Aga Khan University, Harvard University, and the International Training and Education Center for Health (I-TECH). Additional File 1 shows the variety of distance learning programmes that were a part of this assessment.

Additional findings included existence of political will from the government of Tanzania to implement distance learning as a way to solve the challenges of health care worker training, enthusiasm among current distance learning students, and a demand for more and expanded distance learning programmes from health care workers who want greater opportunities and easier access to training.

### Benefits of distance learning programmes for health care workers

Several benefits of distance learning were found, including the ability of distance learning students to continue to work in their facilities and provide for their families while studying. Survey respondents stated that usually, in order to study, there is a need to leave the family due to distant geographic location of the health training institutions. With distance learning, students can stay at home within their own communities. One student noted: "I could not upgrade myself if I could not continue working to support my family. Distance learning is my only choice." One distance learning tutor concurred, noting that "uprooting the learner creates a vacuum, not only in their workplace, but in their family and community."

Several students surveyed mentioned the flexibility of the distance learning programme in that they can study from home around their work schedules. Another health care worker considering enrolling in a distance learning programme said that he believed distance learning could meet his training needs for three reasons: "1) It is cost-effective, as it will be taking place at my physical location (no accommodation, food and transport costs). 2) It is productive, as I will be continuing to do my daily job and other private activities. 3) It is socially-effective, as I will be with my family as usual."

The assessment also found that distance learning helped to limit the indirect costs of training health care workers, i.e., the absence from the health care facility and the burden that places on an already over-burdened system. A MoHSW representative agreed that "[Distance learning] is a better option than always having health care workers leaving their working stations for training." This point was echoed by participants in a focus group discussion as well, who emphasised that there is so much training targeting health care workers, particularly the lower cadres, that they are gone from their facilities for days to weeks at a time.

The surveys of health care workers conveyed positive feedback from prospective distance learning students, who frequently mentioned the importance of being able to continue working while studying, thereby decreasing the strain on human resources for health that already exists in Tanzania. One respondent aptly explained why it is so critical for health care workers to remain in the workforce to the extent possible while studying: "...the region or nation at large has a shortage of about 70% (only 30% staff available), and thus if distance learning will be applied, the staff will continue working, avoiding paralysis of the facility."

Health care workers are required and want to upgrade their skills, but few opportunities exist. There are a limited number of health training institutions, with a limited number of slots for students, and the demand for studying both as new health care worker students and for upgrading, is great. Access to training opportunities is increased with the presence of distance learning programmes. Several students reported that distance learning programmes were a better option than residential programmes which fill up quickly, because there is better access with distance learning programmes; they are "easier to get into".

Distance learning also provides a creative solution to increasing the health care workforce. Converting existing residential upgrading programmes to distance learning upgrade programmes increases both the residential and classroom space available at health training institutions for pre-service training. One health training institution principal said, "Because of MMAM, we must increase enrolment but have no increase in funds for enrolling more students. So, we need to make distance learning programmes successful." Health training institutions can also maximize their space by hosting distance learning students for face-to-face sessions only. One site representative noted that space on campus to hold classes is limited, and having distance learning students come only a couple of times a month for classroom-based sessions helps alleviate this burden. A staff member from one of the health training institutions visited said, "Our resident housing is full; if we do not provide a distance learning track, we will not be able to increase our enrolment."

### Technological feasibility

The assessment found that programmes use a variety of technologies. Of the 25 programmes visited, 21 used some type of distance learning technology. Of these 21, the majority used print-based media (13 programmes). The other programmes in Tanzania utilised computer-based (5), web-based (8), mobile device (3) and videoconferencing (1) technologies, and some programmes used more than one of these technologies. In addition, few programmes used only distance learning technology; 15 programmes used a blended approach, where distance activities were combined with face-to-face sessions and sometimes a practicum component. This approach is common with health care worker training given the need for clinical skills-building,

Although print-based media was most common given its low technology requirements, constraints do exist for print modality, including minimal availability of printed course materials for students due to financial constraints, and problems disseminating materials due to cost and unreliability of the postal service. This finding is supported in the literature. Knebel's review of over 100 distance learning articles found that the portability of print-based modules is especially important to rural learners with limited access to advanced technology. Print materials are generally the cheapest of all the distance learning technologies, and are typically learner-controlled, which is both positive and negative, as they require higher motivation on the part of the learner to complete [13].

Computer and internet based distance learning programmes face more serious constraints related to students' poor computer access and limited computer skills, high cost and slow speed of internet access, inadequate infrastructure, and uneven and unreliable electricity coverage. However, the computer and internet programmes that did exist provided exposure to technology that gave students upgraded computer skills.

Mobile phone technology was found to offer increasing potential for training health care workers, especially in the absence of computers and internet access for students. One organisation was experimenting with sending quizzes and learning content to students via mobile phones.

### Challenges and constraints of distance learning programmes for health care workers

Distance learning in Tanzania faces many challenges and constraints. Resources are inadequate, including funding, space for face-to-face sessions, equipment, and course materials. Just as the HRH crisis impacts health care services, personnel shortages (instructors, preceptors, coordination staff, and IT staff) also impact distance learning programmes. And there is a lack of training and orientation in distance education methodology for instructors and preceptors, as well as inadequate support of distance learning students. This support includes financial support, orientation to the distance learning modality, regular feedback on performance, adequate time with instructors, English language skills training, computer skills training and access, and employer support to study.

Bureaucratic impediments inhibit effective planning and coordination of national distance learning programs. This is mainly due to the centralised structure of the CDE and lack of coordination across the districts. National programmes lack guidelines and specification of competencies for programme managers, tutors, and preceptors to clarify their roles and responsibilities.

In some programmes, a disconnect between theory and the practical structure exists. While curricula for the national distance learning programmes are based on Tanzanian national clinical guidelines and created for the Tanzanian context, other programmes use curricula or materials developed outside the country (in other parts of Africa or Europe). According to students who use the latter, these curricula and materials lack grounding in the Tanzanian context. Additionally, some curricula for national programmes are outdated, and severe material shortages existed in every programme assessed. It was common for dozens of students to share one study module, or for students to wait several months to receive printed modules in the mail.

According to several tutors interviewed, poor English language skills (especially writing) are sometimes a barrier to students' learning effectively through distance learning. Although English is the official language of instruction in Tanzania starting at the secondary school level, programme staff and tutors commented that this lack of English-language proficiency is attributable to the fact that the Tanzanian educational system is not as developed as others in the region. One preceptor interviewed for the study noted about the materials provided by the CDE:

"... the fact that they are in English is a barrier--the students for this program only have a primary education besides their MCHA [Maternal Child Health Aide] training. It would be better if the materials and the training were in Kiswahili but it is not allowed. That is one reason students need more classroom time, in order that an instructor can translate the materials for them and explain them in Swahili."

Finally, none of the upgrade programmes currently being implemented had monitoring and evaluation plans in place to track and report on programme completion rates, to assess students' on-the-job performance, or to track location after graduation. Most programmes assessed students through tests and assignments.

### Financial feasibility

Related to student financial support, several studies have shown that distance learning can be cost effective [13,17]. This assessment found a comparison done by MoHSW of distance learning and residents courses (MoHSW: Distance education training needs assessment and unit cost study: A mini-study, May-June 2007, unpublished report). The CDE conducted a cost analysis of the clinical assistant to clinical officer distance learning upgrade programme compared with the corresponding residential training upgrade programme. It found that the cost to the MoHSW for maintaining one distance learning student for one year is TSH 172 000 (US$ 143), compared with TSH 300 000 (US$ 250) for one student for one year for the residential course. However, this cost analysis was cost per student as opposed to cost per graduate, and a programme with a high drop-out rate would be less cost effective.

All programme managers interviewed said that distance learning upgrade programmes are less costly than residential upgrade programmes, requiring fewer tutors, less classroom space and equipment, and lower housing and food costs for students. One programme manager said, "The students are not present on-site the entire length of the programme (only during face-to-face sessions), so we pay less costs for them than for our residential students."

However, there are several hidden costs to a distance learning programme, including high start up costs and increased workload to instructors, as well as additional costs borne by the students. In Tanzania, tuition is often covered by the government but students also had travel and accommodation costs for face to face meetings with instructors, costs to use internet cafes, and costs for printing and photocopying of modules and additional resource materials.

### Distance learning to address health care worker shortages

In terms of using distance learning to increase the number of health care workers, the assessment did not find any distance learning pre-service programmes in Tanzania. The distance learning programmes reviewed mainly aimed at increasing the skills and qualifications of existing health care workers. Literature reviews by Knebel [13] and Mattheos [17] had similar results, finding few distance learning applications for health care undergraduates. Results suggested that pure distance learning is not an appropriate modality for pre-service training of health care workers, given the life-and-death nature of the work, the hands-on, practical skills orientation of health care worker training, and the need for socialization and integration into academic life of new students. In Tanzania, this sentiment was echoed by a MoHSW official and a tutor, as well as some participants of the focus group with care and treatment partners. They believed distance learning for health care workers is best applied to in-service upgrading and continuing education participants rather than pre-service education, stating that the former are more adequately prepared and better able to be self-directed because of their work experience.

However, distance learning upgrading programmes do help the MoHSW to retain its current staff by providing opportunities for professional development. Several students interviewed in upgrade programmes stated they intended to continue working in their current health facilities, and expected to be promoted. Also, by providing upgrading opportunities through distance learning rather than in residential health training institutions, more pre-service slots were then made available at the crowded health training institutions for new students. Finally, health care workers were able to remain at their work place while undergoing further training through distance learning, thus ensuring that upgrading health care workers is benefitting rather than creating further burdens on the system.

### Limitations

Several limitations to this assessment exist. Although the assessment aimed to be a comprehensive assessment of all distance learning programmes for health care workers in Tanzania, the authors cannot be sure that every programme was found and assessed. The study did include all distance learning programmes known to the MoHSW and its partners, as well as those additional programmes identified by the distance learning programmes themselves during interviews.

In addition, the assessment did not compare the quality of clinical work of health care workers who had graduated from distance learning programmes with those who had graduated from comparable residential programmes; nor did it assess the quality of teaching or learning materials in relation to residential programmes' materials. These remain limitations to determining the true viability of this modality. There was anecdotal evidence from our assessment that distance learning students, on average, performed as well as residential students in the qualifying exam for their cadres, but we do not know how this translates into clinical practice. This would be a useful area for further research.

In addition, the study did not attempt to compare Tanzania's distance learning offerings with those of other countries. Programmes from other countries were looked at to garner lessons learned about what factors contribute to a successful distance learning programme, and it is hoped that these lessons may be applied to improving programmes in Tanzania. A regional comparison of programmes would be an interesting future study.

## Discussion

Distance learning is a viable method for increasing the skills of health care workers in low-resource settings. It offers several advantages: students can continue to work at their health facilities while they are upgrading, thus continuing to support themselves and their families, and also ensuring that their health facilities do not experience staffing challenges as a result of the health care workers' participation in training.

A low-tech approach is particularly feasible in developing countries like Tanzania, utilizing print-based materials to reach health care workers in rural settings with poor infrastructure. Lower-end internet-based videoconferencing, and the use of flash drives and CD-ROMs are effective in areas where there is computer and internet access, but it is important to note that technology should not be used for its own sake. It should be appropriate to the goals, learning tasks and setting of the distance learning programme. Given the need for hands-on, skills-based learning in health care worker training, a blended approach that combines face-to-face sessions with instructors and other learners, a practical/clinical component, and self-directed study (at a distance) is most effective.

Distance learners need support and continuous monitoring to succeed at the programme. To provide this support and coverage nationwide, a decentralized set-up is important, preferably within an existing structure of the MoHSW.

It is also important to build in a strong orientation to distance learning for students at the start of their study, including sessions on good studying skills and guidance on self-directed learning, as this aspect of the educational process is very different from a traditional educational setting in Tanzania. It is also important to build in an option for a learning community right from the beginning, linking distance learning students to others in their geographic area to create support/study groups. This will lessen the isolation that many distance learning students feel which can lead to dropping out. Because of this need for self-directed learning, distance learning may not be the most appropriate approach for students new to the health care profession in a pre-service training programme.

Distance learning programmes require appropriate training materials, developed specifically for the distance learning modality, and tutors and preceptors need specialised training before teaching in a distance learning programme.

Some of the findings of our assessment are unique to the Tanzanian context, while others are common in the region. In comparison to other countries where similar assessments were conducted [19,20], distance learning in Tanzania is recognized as an important strategy by the MoHSW and is included in key policy documents and strategic plans. This commitment is evidenced by the creation of a centre specifically for training health care workers via distance learning (the MoHSW Centre for Distance Education). However, a similar assessment conducted in Mozambique [19] shows the same infrastructure challenges, lack of computer skills, human resource challenges, and other feasibility issues. Greater resourced countries in the region, such as South Africa, may align more with the results of an assessment done in Trinidad and Tobago [20] which show more widespread access to computers and internet by health care workers; more affordable high speed internet services for individuals and institutions; internet and computer labs in national health training institutes; use of videoconferencing and e-learning; in-country expertise to develop and implement distance learning programmes; greater teaching capacity in distance learning modalities; and trained IT specialists able to support distance learning technologies. In relation to this, two distance learning staff interviewed for this assessment had travelled to South Africa to learn from its distance learning programmes and recommended South African tutors come to Tanzania to train local tutors. Capacity in Tanzania may be strengthened by leveraging the expertise of South Africa and other similarly-resourced countries.

## Conclusions

Distance learning programmes hold great potential to increase the motivation, knowledge, and skills of Tanzania's current health care workforce, and properly planned pre-service programmes, utilizing distance learning as one component, may also contribute to reducing the country's shortage of health care workers. This assessment revealed that distance learning programmes in Tanzania have achieved great success but face numerous challenges and constraints; however, if resources for distance learning are increased and if stakeholders commit to collaborating across programmes to share best practices and lessons learned, existing programmes can be improved and new programmes developed. Accomplishing this is critical in retaining current health care workers and increasing the skills and the numbers of qualified health care workers capable of meeting the health care needs of the Tanzanian population.

## Competing interests

DK is director of the Tanzania MoHSW Centre for Distance Education.

## Authors' contributions

AN, LS, AS, KP, and MK contributed to the conception, design and methodology of the assessment. AN, MK, DK and LS carried out the fieldwork. AN and KP analysed the data and drafted the initial assessment report. AS, LS, MK, and DK contributed to the final report. AN and LS drafted the article manuscript, and KP drafted the methods section. All authors read and approved the final manuscript.

## Supplementary Material

Additional File 1 **List of Programmes Surveyed for the Assessment**. This file contains a table of all the programs that were visited, contacted or reviewed for the distance learning assessment conducted in Tanzania. Click here for file
