# Supplementary material for: Increasing health worker capacity through distance learning: a comprehensive review of programmes in Tanzania
Source: Hum Resour Health. 2010 Dec 31;8:30. doi: 10.1186/1478-4491-8-30 (PMC3023774; doi:10.1186/1478-4491-8-30)
Supplement: Additional File 1 — List of Programmes Surveyed for the Assessment. This file contains a table of all the programs that were visited, contacted or reviewed for the distance learning assessment conducted in Tanzania. [file 1478-4491-8-30-S1.DOCX]

**Additional File 1: List of Programmes Surveyed for the Assessment**

| **Institution/Programme** | **Description** | **Place** |
| --- | --- | --- |
| **Site Visits to the Centre for Distance Education (CDE), Clinical Assistant Training Centres (CATCs), Clinical Officer Training Centres (COTCs), Zonal Health Resource Centres (ZHRCs), and Zanzibar Ministry of Health and Social Welfare (MoHSW)** | | |
| Centre for Distance Education, Morogoro*^a^* | National MoHSW centre for distance education for health care workers in Tanzania; various print-based, national upgrade programmes for health care workers, including current programmes (clinical assistant [CA] to clinical officer [CO], maternal and child health aid [MCHA] to enrolled nurse [EN], and EN to registered nurse [RN]) in collaboration with Aga Khan University (AKU); and planned programmes (CO to assistant medical officer [AMO], laboratory assistant to laboratory technician, health assistant to health officer, and pharmacy assistant to pharmacy technician) | Morogoro, Tanzania |
| CDE, Morogoro: Collaboration with Cardiff University, UK, on a Sexually Transmitted Infection (STI) Module*^b^* | STI print-based module for planned 2‑year national upgrade programme for CO to AMO; coordinated from CDE | Morogoro, Tanzania |
| CDE, Morogoro: MCHA to EN Upgrade Programme*^a^* | Print-based, 2-year national upgrade programme; coordinated from CDE | Morogoro, Tanzania |
| Maswa CATC: CA to CO Upgrade Programme*^a^* | Print-based, 3-year national upgrade programme; coordinated from CDE | Maswa, Tanzania |
| Kigoma CATC: CA to CO Upgrade Programme*^a^* | Print-based, 3-year national upgrade programme; coordinated from CDE | Kigoma, Tanzania |
| Kilosa COTC: CA to CO Upgrade Programme*^a^* | Print-based, 3-year national upgrade programme; coordinated from CDE | Kilosa, Tanzania |
| Centre for Educational Development in Health Arusha (CEDHA)/Northern ZHRC: Diploma in District Health Management for Health Managers and Health Workers in the Districts*^a^* | Print-based, 3-year upgrade programme; coordinated from Northern ZHRC in four zones | Arusha, Tanzania |
| CEDHA/Northern ZHRC: e‑Collaboration Project using Global Campus 21 in collaboration with Capacity Building International (InWEnt), MoHSW, and ZHRCs*^a^* | Information and communication technology (ICT) activity aimed at creating a method for distance communication across ZHRCs and the MoHSW; coordinated from Northern ZHRC | Arusha, Tanzania |
| Eastern ZHRC, Morogoro*^a^* | No distance learning activities at this time; shares same infrastructure as CDE | Morogoro, Tanzania |
| Lake ZHRC, Mwanza*^a^* | No distance learning activities at this time; collaborates in a limited capacity with the distance learning CA to CO programme in Maswa | Mwanza, Tanzania |
| Western ZHRC, Kigoma*^a^* | No distance learning activities at this time; collaborates in a limited capacity with the distance learning CA to CO programme in Kigoma | Zanzibar, Tanzania |
| Zanzibar MoHSW Continuing Education Unit (CEU)*^a^* | Assists in coordinating AKU/CDE national EN to RN upgrade programme for Zanzibar students; developing infrastructure to enable additional ICT programmes | Zanzibar, Tanzania |
| **Site Visits and Reviews of Distance Learning Programmes in Tanzania** | | |
| Aga Khan University; Tanzania Institute for Higher Learning Advance Nursing Studies Programme in Dar es Salaam: EN to RN*^a^* | Print-based, 2-year upgrade programme; coordinated in collaboration with CDE out of AKU in Dar es Salaam; aimed at students in Dar es Salaam, Zanzibar, and Morogoro | Dar es Salaam, Tanzania |
| Aga Khan University; Advance Nursing Studies Programme in Zanzibar: EN to RN*^a^* | Print-based, 2-year upgrade programme coordinated out of AKU in Dar es Salaam; additional coordination for Zanzibar students from the Zanzibar MoHSW CEU | Zanzibar, Tanzania |
| AHADI Institute*^a^* | Serves as centralised coordinator of distance learning programmes from accredited educational institutions in Europe; aimed at youth in the Africa Great Lakes Region; programmes include law degree, social welfare diploma, teacher training degree, and economics degree | Kigoma, Tanzania |
| Harvard University HOPE (HIV Online Provider Education) webcasts*^a^* | Live, bi-monthly internet-based presentations/discussions from faculty presenters on HIV-related topics, aimed mainly at health care providers at various Harvard University–funded project sites around the globe | Dar es Salaam, Tanzania |
| Ifakara Health Institute, Ifakara*^a^* | No distance learning activities at this time, although strong technological infrastructure; utilises personal digital assistants (PDAs) in research programme | Ifakara, Tanzania |
| International Institute for Communication and Development (IICD)*^a^* | Implementing various ICT projects in a wide range of fields throughout Tanzania; implementation of a District Health Management Information System (HMIS) in eight hospitals and four faith-based organisations (FBOs); conversion of conventional in-service and pre-service courses and workshop materials into e-learning courses for use by Muhimbili University of Health and Allied Sciences (MUHAS) | Dar es Salaam, Tanzania |
| InWEnt, Global Campus 21*^a^* | InWEnt provides various e-learning courses in the areas of health, education, business, and humanitarian studies through a programme called Global Campus 21; participants include MoHSW, Ministry of Education (MOE), and MUHAS personnel and students; utilised by Northern ZHRC for staff and students | Dar es Salaam, Tanzania |
| MUHAS*^a^* | Various electronic resources available to students; internet portal providing health information to and from the districts, regions, health experts, and postgraduate students | Dar es Salaam, Tanzania |
| Open University of Tanzania (OUT)*^a^* | Several print- and internet-based distance learning degree programmes in science, education, business management, and law; implemented through 25 Regional Centres and 69 Study Centres | Dar es Salaam, Tanzania, and other areas |
| Phones for Health*^a^* | Mobile phones used to transmit data to and from health care workers, mainly for disease reporting and surveillance | Dar es Salaam, Tanzania |
| Tanzania Education and Research Network (TERNET)*^c^* | Network of higher-learning and research institutions connected through high-speed internet using ICT resources to enhance research and education in Tanzania and facilitate e-learning activities | Throughout Tanzania |
| Tanzania Global Development Learning Centre (TGDLC)*^c^* | Non-profit organisation that offers high-end videoconferencing using a wideband satellite system linked to more than 120 global conferencing centres; e-learning courses and a computer lab with Internet capabilities and CD-ROM, print, and video services are available for fees; for use by public and private sectors | Dar es Salaam, Tanzania |
| World Health Organisation (WHO): Integrated Management of Childhood Illness Computerised Adaptation and Training Tool (ICATT): Dar es Salaam*^a^* | E-learning software application developed as an alternative training approach to support implementation of the WHO/UNICEF strategy on the Integrated Management of Childhood Illness (IMCI); includes clinical guidelines, chart books/algorithms, and resources | Dar es Salaam, Tanzania |
| **Conferences Attended in Tanzania** | | |
| Second National Telemedicine Conference*^a^* | Eighteen attendees convened to capture lessons learned, discuss overcoming challenges and barriers, identify practicing organisations and institutions, and provide input for the development of a national telemedicine policy | Dar es Salaam, Tanzania |
| mHealth Workshop: Mobile Phone Applications for Public Health*^a^* | Fifty participants gathered to present and discuss application and implementation of mHealth, including use of mobile devices for outreach, telemedicine, data gathering, and clinical care | Dar es Salaam, Tanzania |
| **Visits to Tanzania Information Technology (IT)/Distance Learning Technology Organisations** | | |
| AIM Consultants*^a^* | IT services company providing comprehensive IT support and services to small and medium-sized businesses, individuals, and governmental and non-governmental organisations | Dar es Salaam and Arusha regions of Tanzania |
| MoHSW IT Unit*^a^* | Provides IT support services to other units of the MoHSW; responsible for installation of computers and very small aperture terminals (VSAT) at all ZHRCs; provides user support, develops custom software (if needed), maintains MoHSW website, and provides ICT inputs | Dar es Salaam, Tanzania |
| Soft-Tech*^a^* | One of the largest IT and telecommunications service providers in Tanzania; offers IT training, telecommunications services (including digital videoconferencing and satellite), and banking and ATM services | Dar es Salaam, Tanzania |
| **Review of Distance Learning Programmes with Contexts Similar to that of Tanzania** | | |
| Africa Teledermatology Project*^c^* | Uses web-based telemedicine technology to provide medical services at a distance by connecting several medical centres to enable exchange of expert dermatology information; aimed at local physicians, dermatologists, and health care workers in hospitals and clinics throughout Africa | Uganda, Botswana, Malawi, Swaziland, Burkina Faso, and Lesotho |
| African Medical and Research Foundation (AMREF)*^c^* | Print-based and e-learning upgrade programmes for nurses | Based in Kenya with offices in Uganda, Ethiopia, South Africa, Sudan, and Tanzania |
| Caribbean Health Leadership Institute (CHLI) and University of West Indies (UWI)*^b^* | Year-long e-learning course to train health professionals in leadership | Jamaica |
| HIV [e]Ducation*^b^* | Three-month-long e-learning programme utilising memory sticks for training health care workers in HIV | Caribbean, India, Mozambique, Netherlands, Uganda, Kenya, Tanzania, Malawi, and Indonesia |
| JHPIEGO, Distance Learning Course for Antiretroviral Therapy (ART) Providers*^c^* | Ten-week distance learning course on ART and management of opportunistic infections for HIV and AIDS for ART care and treatment providers, including physicians, COs, nurses, pharmacists, lab technicians, and environmental health technologists | Zambia |
| Johns Hopkins Centre for Clinical Global Health Education (CCGHE) and Global Health eLearning Centre*^c^* | Live infectious disease grand rounds webcasts and videoconferencing; HIV clinical care discussions; online resource for clinicians; e-learning courses; master in public health distance learning degree programme | US-based/global (India, Ethiopia, Tanzania, Zambia, Democratic Republic of the Congo, and Uganda) |
| Learning for International Non-Governmental Organisations (LINGOs)*^b^* | Members have access to e-learning courses, e-learning software, and language training | US-based/global |
| Mildmay Centre*^b^* | In conjunction with Manchester University in the UK, offers a modular diploma and degree in HIV programme management and is part of a bachelor of science degree programme titled ‘Health Systems Approach to HIV and AIDS Care and Management’ | Uganda, Kenya |
| Mindset*^c^* | Distance learning is offered to health care workers and the general public through satellite broadcast, a website, and videos | South Africa |
| Réseau en Afrique Francophone pour la Télémédecine (RAFT)*^b^* | Webcasting sessions for clinicians and hospital administrators in 15 West African countries; internet-based telemedicine/teleconsultations | West Africa region |
| University of Swaziland Distance Learning*^c^* | Various web-based distance learning courses and bachelor degrees | Swaziland |
| Videoconferencing at three South African Universities (Stellenbosch, University of Pretoria, and the University of Freestate)*^c^* | Videoconferencing sessions presented radiology topics | South Africa |
| **Review of Distance Learning Programmes in the I-TECH Network** | | |
| I-TECH HIV/AIDS Clinical Seminar Series*^b^* | Bi-monthly web-based distance learning series aimed at health care workers treating HIV and AIDS in Africa, the Caribbean, and India; HIV and AIDS experts present on a variety of advanced care, comprehensive management, and treatment topics via live sessions across several countries | Seattle; Africa region, Caribbean region, India |
| I-TECH Botswana Monitoring and Evaluation Officer Mentoring Programme (District Level)*^b^* | Three-year programme that combines national training, regional training, on-site mentoring, and self-study exercises to be completed by mail | Botswana |
| I-TECH Global Staff In-Service Series*^b^* | Live, web-based sessions on a variety of professional development topics aimed at staff within the I-TECH network | Seattle and I-TECH global network |
| I-TECH India: Clinical Consultation Warmline*^b^* | Phone line devoted to answering clinicians’ questions concerning HIV and ART, post-exposure prophylaxis (PEP), and perinatal care to support clinicians across India in providing quality HIV and AIDS treatment and care | India |
| I-TECH India: HIV Listserv*^b^* | Monthly listserv updates on HIV and AIDS for clinicians | India |
| I-TECH Namibia Digital Videoconferencing (DVC) Project*^b^* | Use of videoconferencing for meetings, demonstrations, film forums, and presentations aimed at Ministry of Health clinicians and staff | Namibia |
| University of California, San Diego’s (UCSD) Clinical Case Teleconference with I-TECH South Africa*^b^* | Live, on-line, weekly teleconference hosted by UCSD’s I-TECH mentors; review of patient cases related to HIV, along with open discussion with clinical mentors from South Africa, Peru, Mexico, and the US | Eastern Cape, South Africa |
| *^a^*Programmes, sites, or conferences that assessment teams observed, attended, or interviewed in person.  *^b^*Programmes that were interviewed via phone or e-mail questionnaire.  *^c^*Programmes for which websites, programme reports, and published articles were reviewed. | | |
